# Pyroptosis: a new paradigm of cell death for fighting against cancer

**DOI:** 10.1186/s13046-021-01959-x

**Published:** 2021-05-03

**Authors:** Yixin Tan, Quanzhu Chen, Xiaoling Li, Zhaoyang Zeng, Wei Xiong, Guiyuan Li, Xiayu Li, Jianbo Yang, Bo Xiang, Mei Yi

**Affiliations:** 1 NHC Key Laboratory of Carcinogenesis, Hunan Provincial Cancer Hospital and the Affiliated Cancer Hospital of Xiangya School of Medicine, Central South University, Tongzipo Road, Changsha, 410013 Hunan China; 2 The Key Laboratory of Carcinogenesis and Cancer Invasion of the Chinese Ministry of Education, Cancer Research Institute and School of Basic Medical Sciences, Central South University, Changsha, 410078 Hunan China; 3 Hunan Key Laboratory of Nonresolving Inflammation and Cancer, The Third Xiangya Hospital, Central South University, Changsha, 410013 Hunan China; 4 Department of Dermatology, The Second Xiangya Hospital, The Central South University, Changsha, 410011 Hunan China; 5 Department of Laboratory Medicine and Pathology, University of Minnesota, Minneapolis, MN 55455 USA; 6 Department of Dermatology, Xiangya Hospital, The Central South University, Changsha, 410008 Hunan China

**Keywords:** Tumor microenvironment, Gasdermin, Necroptosis, Ferroptosis, Immune checkpoint, Adaptive immunity, Immunogenic cell death

## Abstract

**Background:**

Unraveling the mystery of cell death is one of the most fundamental progresses of life sciences during the past decades. Regulated cell death (RCD) or programmed cell death (PCD) is not only essential in embryonic development, but also plays an important role in the occurrence and progression of diseases, especially cancers. Escaping of cell death is one of hallmarks of cancer.

**Main body:**

Pyroptosis is an inflammatory cell death usually caused by microbial infection, accompanied by activation of inflammasomes and maturation of pro-inflammatory cytokines interleukin-1β (IL-1β) and interleukin-18 (IL-18). Gasdermin family proteins are the executors of pyroptosis. Cytotoxic N-terminal of gasdermins generated from caspases or granzymes proteases mediated cleavage of gasdermin proteins oligomerizes and forms pore across cell membrane, leading to release of IL-1β, IL-18. Pyroptosis exerts tumor suppression function and evokes anti-tumor immune responses. Therapeutic regimens, including chemotherapy, radiotherapy, targeted therapy and immune therapy, induce pyroptosis in cancer, which potentiate local and systemic anti-tumor immunity. On the other hand, pyroptosis of normal cells attributes to side effects of anti-cancer therapies.

**Conclusion:**

In this review, we focus on the regulatory mechanisms of pyroptosis and the tumor suppressive function of pyroptosis. We discuss the attribution of pyroptosis in reprogramming tumor microenvironments and restoration of anti-tumor immunity and its potential application in cancer immune therapy.

## Background

Cell death is one of the most fundamental issues of life. As a hallmark of cancer, the ability to escape cell death not only contributes to the origin of cancer, but also plays an essential role in acquisition of therapy-resistance, relapse and metastasis [1]. The ultimate goal of cancer therapeutics, including radiotherapy, chemotherapy, and immunotherapy that has recently made great achievements, is to maximize the destruction of tumor cells, but minimize the damage to normal tissues. However, the inherent genetic and epigenetic heterogeneity of tumor cells, as well as metabolic plasticity and other factors, confer tumor cells a greater adaptability to the unfavorable tumor environments, resulting in acquisition of therapy resistance and metastatic potential. Cell death is generally categorized as regulated cell death (RCD) or accidental cell death (ACD). ACD is referred to a biologically uncontrolled cell death or non-programmed cell death which usually presents as lytic or necrotic like form, whereas RCD is a genetically controlled process. Necrotic cell death has been considered merely as a non-programmed cell death for a long time. However, now we clearly know that necrotic like cell death can be executed in a finely controlled manner [2]. During the past decades, characterizations of new forms of RCD and exploration of its roles in physiological or pathological conditions have deepened our understanding on inflammation, immunity and cancer development.

The anti-tumor strategy has now switched from killing the entire tumors barely through drugs or radiation to achieving long-term control of cancer by eliminating residue malignant cells through the body’s inherent immune mechanism. The death of tumor cells may be immunogenic or non-immunogenic. Induction of immunogenic cell death (ICD) of tumor cells is prerequisite for rebuilding anti-tumor immunity. ICD refers to cell death that generates adaptive immunity against endogenous or exogenous antigens carried by dying cells [3]. The most essential nature of ICD is the complex cell-to-cell communications between immune cells and dying cells [4]. The key parameters that determine the immunogenicity of cell death include antigenicity, inflammation and adjuvanticity [5]. Dying cells undergo lytic death, providing dendritic cells (DC) with antigen and inflammatory stimuli, and then activate CD8^+^ T cells through a process called antigen cross-priming [6]. ICD was initially identified as a protective mechanism against pathogen infection. Pathogen-infected cells release pathogen-related molecular patterns (PAMPs) that are conserved microbial molecules which could be recognized by pattern-recognition receptors (PRRs) of the innate immune system to initiate PAMP-triggered immunity [4]. Sterile ICD can be induced by chemotherapy [7]. In ICD induced by chemotherapy or radiotherapy, dying cells release damage-associated molecular patterns (DAMPs), also known as alarmin, which may initiate and exacerbate the immune response through corresponding PRRs on immune cells [8, 9].

**Fig. 1:**
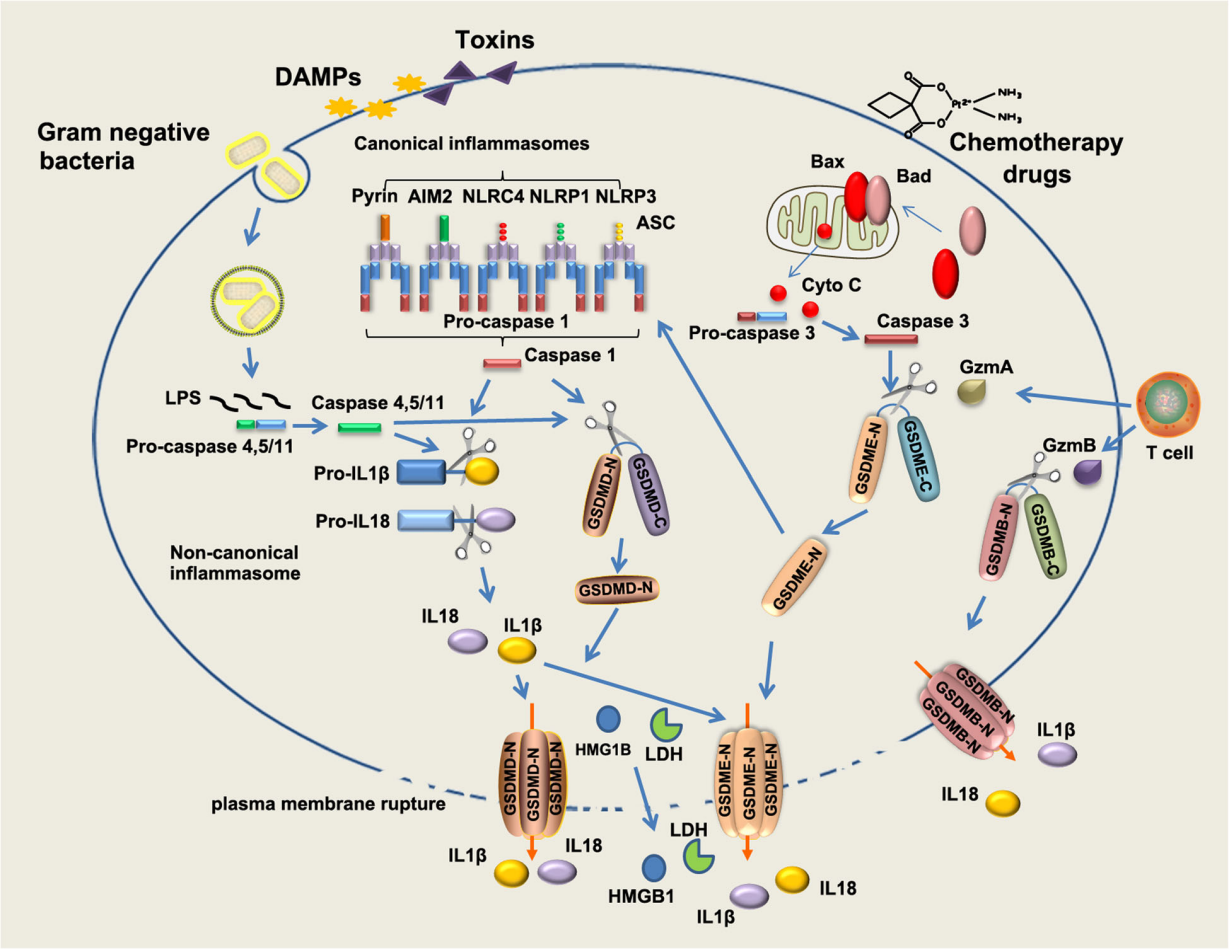
The canonical inflammasome and non-canonical inflammasome pathway in pyroptosis. The canonical inflammasome is assembled in response to exogenous pathogens and endogenous damage by intracellular sensor proteins, including NLRP1b, NLRC4, NLRP3, AIM2 and Pyrin. The canonical inflammasomes recruit pro-caspase 1 through inflammasome adaptor protein ASC, leading self-cleavage and activation of caspase 1. Active caspase 1 cleaves pro-inflammatory cytokines pro-IL-1β, pro-IL-18, leading to maturation of IL-1β, IL-18. Active caspase 1 cleaves GSDMD protein at the middle linker, liberating the cytotoxic N-terminus to form pore on plasma membrane, which allows the release of mature IL-1β, IL-18. In non-canonical pathway, LPS directly binds to murine pro-caspase 11 or its human homologs pro-caspase 4 and 5, leading activation of caspase 11/4/5. In non-canonical inflammasome pathway, cleavage of GSDMD is executed by active caspase 11 or caspase 4 and 5 upon direct binding of cytosolic LPS. Chemotherapy drugs could induce pyroptosis in epithelial cells through activating mitochondrial death machinery and caspase 3. In this case, GSDME is cleaved by active caspase 3. GSDME-N in turn activates NLRP3 inflammasome, leading to activation of caspase 1/GSDMD cascade, which promotes maturation of IL-1β, IL-18. Gasdermins could be cleaved by Lymphocyte-derived granzymes proteases, unleashing the pore-formation ability to trigger pyroptosis of cancer cells

The discovery of new forms of ICD and their roles in immunity and tumorigenesis have promoted the renewal of anti-tumor treatment strategies. Pyroptosis is a newly characterized form of ICD and has gradually emerged as a great opportunity to improve the efficacy of cancer immune therapy. Pyroptosis usually occurs in macrophage upon pathogen infection. It plays an essential role in clearance of pathogens [10]. Morphologically, pyroptosis is featured by cell swelling and plasma membrane rupture, leading to release of pro-inflammatory cytokines IL-1β, IL-18 and cellular contents into the extracellular space and activating inflammatory response (Fig. 1). Mitochondria remain intact and there is no leakage of cytochrome C during pyroptosis in macrophage [11, 12]. Epithelial cells also undergo sterile pyroptosis in physiological or pathological conditions. For example, pyroptotic cell death of intestinal epithelial cells mediated by caspase-1 activation is a cause of mucosal barrier dysfunction in Crohn’s disease [13]. Sterile pyroptosis also occurs in epithelial cells upon various death stimuli, including anti-neoplastic drugs [14, 15]. Pyroptosis in epithelial cells could occur at downstream of the mitochondrial apoptotic pathway [16]. As a highly-immunogenic form of cell death, pyroptosis causes local inflammation and attracts inflammatory cell infiltration, providing a great opportunity to relieve immunosuppression of tumor microenvironments (TME) and induce a systemic immune response in treating solid tumors [17].

## Main text

To date, there are four distinct pathways were identified to induce the action of pyroptosis. Basically, pyroptosis could be executed in an inflammasome dependent or independent manner. The inflammasome dependent pathways include the canonical and non-canonical pathways, whereas the independent pyroptosis pathways include caspase-3 mediated pathway and granzymes proteases mediated pathway. We will discuss in detail in the following section.

### The canonical inflammasome pathway

Pyroptosis can be induced by the canonical caspase-1 inflammasome or directly activated by caspase-4, − 5 and − 11 bound with lipopolysaccharide (LPS) (Fig. 1). Inflammasomes are multi-protein complexes assembled in response to microbial infections, pathogen associated molecular patterns (PAMPs) and endogenous DAMPs [18]. It has been widely recognized that inflammasome play a central role in the innate immune pathways [18]. Assembly of canonical inflammasome is initiated by sensing exogenous pathogens and endogenous damage, including bacterial infection, cytosolic double-stranded DNA (dsDNA), crystals and toxins, by a range of intracellular sensor proteins, including NOD-like receptors (NLRP1b, NLRC4 and NLRP3), a member of the HIN200/AIM2-like receptor family (AIM2) and a member of TRIM family (Pyrin/TRIM20) (Fig. 1). Upon intracellular recognition of diverse danger factors, sensor proteins homo-oligomerize and recruit the inflammasome adaptor apoptosis-associated speck-like protein containing a CARD (ASC) through Pyrin domains, bridging inflammasome sensors with pro-caspase-1 and leading to caspase-1 activation through self-cleavage [19, 20]. The gasdermin D (GSDMD) was identified to be a direct substrate of inflammatory caspases and function as a major executor of pyroptosis in macrophages [14, 21, 22]. Active caspase-1 or caspase-11 cleaves GSDMD at its middle linker, liberating the gasdermin-N domain to form pore on plasma membrane. Active caspase-1 also cleaves pro-inflammatory cytokines IL-1β, IL-18, leading to maturation of IL-1β, IL-18 [14, 22–24] (Fig. 1). In addition to inflammasome-activated caspase-1 or − 11, macrophage GSDMD also could be processed by caspase-8 upon TAK1 blockade by Yersinia bacteria [25]. Rapid/excessive pores formation enables release of pro-inflammatory cytokines IL-1β, IL-18 to extracellular environments, leading to immune cells infiltration and establishment of an inflammatory microenvironment [26]. Pyroptosis also contributes to release of DAMPs such as the protein high-mobility group box 1 (HMGB1) and lactate dehydrogenase (LDH), resulting in amplifying inflammation and recruiting immune cells in the tissue [27–29]. However, the means by which HMGB1 releases to extracellular space is controversial. Though GSDMD is required for secretion of IL-1β and HMGB1 following inflammasome activation, GSDMD pore is not a direct conduit for HMGB1. HMGB1 release after inflammasome activation only occurs when plasma membrane integrity is disrupted [30]. A latest study revealed that ill-characterized nerve injury-induced protein 1 (NINJ1), a transmembrane protein localized at cell surface, is the essential mediator for plasma membrane rupture during pyroptotic cell death. Ninj1^−/−^ macrophages are unable to release HMGB1 and LDH in response to diverse inducers of lytic cell death [31].

### The non-canonical inflammasome pathway

In the non-canonical inflammasome pathway, intracellular LPS binds directly to caspase-4/5/11 via CARD domain and initiates oligomerization of caspase-4/11, leading to activation of the caspases [32] (Fig. 1). In non-canonical inflammasome pathway, cleavage of GSDMD at Asp276 is executed by active murine caspase-11 or its human homologs caspase-4 and -5 upon direct binding of cytosolic LPS [21, 33–35] (Fig. 1). The catalytic domains of inflammatory caspases directly bind to GSDMD and execute cleavage at residues FLTD [36]. A p10 product generated from caspase-4/11 autoprocessing is necessary and sufficient to cleave GSDMD. The p10 fragment of caspase-4/11 binds with GSDMD-C domain, leading to dimerization-mediated caspase activation and cleavage of GSDMD [37]. NLRP3 is also activated by caspase-11 mediated non-canonical inflammasome, thus leading to maturation of IL-1β and IL-18 [38, 39]. Cytosolic LPS or cytosolic Gram-negative bacteria activates non-canonical (caspase-4/11) inflammasome signaling and induces pyroptosis of neutrophil in a GSDMD-dependent manner, leading to extrusion of neutrophil extracellular traps (NETs) [40], thus non-canonical inflammasome links pyroptosis and NETosis, which is a unique type of regulated neutrophil cell death in response to infection of pathogens [41].

### Caspase-3/GSDME mediated pathway

Sterile pyroptosis may occur in epithelial cells. For example, chemotherapy drugs induce pyroptosis in epithelial cells through caspase-3 mediated cleavage of gasdermin E (GSDME) [16, 42] (Fig. 1). Active caspase-3 after TNF-α stimulation cleaves human and mouse GSDME at position 267 or 270 amino acid residue. GSDME mutants in which aspartate at position 267 or 270 was substituted by alanine lose the activity to execute pyroptosis [42]. Neither the canonical nor the non-canonical inflammasome is required for caspase-3/GSDME mediated pyroptosis. However, GSDME-N generated by active caspase-3 could activate the canonical inflammasome pathway and thus promote maturation and release of IL-1β and IL-18 (Fig. 1). During the past decades, activation of caspases, especially caspase-3, was thought to be one of the biochemical features of apoptosis process. Now we know that activation of caspase-3 is not specific to apoptosis. The gasdermins, rather than caspases, is the central switch from apoptosis to pyroptosis upon death stimuli.

### The granzymes mediated pathway

Killer cells mediated elimination of tumor cells is previously considered to be noninflammatory. Recently, studies indicated that natural killer cells and cytotoxic T lymphocytes elicit pyroptosis of cancer cells through granzymes proteases mediated cleavage of specific gasdermin family members [43, 44] (Fig. 1). For example, lymphocyte-derived granzyme A (GZMA) cleaves gasdermin B (GSDMB) at the linker, which unleashes its pore-forming activity and results in pyroptotic cell death of GSDMB-expressing cancer cells [43] (Fig. 1). The granzyme B (GZMB) from natural killer cell or chimeric antigen receptor (CAR) T cell directly cleaves GSDME after D270 residue where the site caspase-3 also cleaves, liberating cytotoxic N-terminus to form pore in membrane [44, 45] (Fig. 1). Granzymes mediated pyroptosis of cancer cells may magnify inflammation signals in TME, thus recruit more immune cells and further ignite antitumor immunity.

### Gasdermin family members, the executioner of pyroptosis

Gasdermin is a family of pore-forming proteins playing an essential role in the execution phase of pyroptotic cell death. Human gasdermin family contains six conserved members, including gasdermin A, B, C, D, E (also named as DFNA5), and DFNB59. Mice do not have gasdermin B, but there are triplicated gasdermin A (gasdermin A1–3) and quadruplicated gasdermin C (gasdermin C1–4) [46]. The gasdermin family members have an autoinhibited two-domain architecture that is consisted of a cytotoxic N-terminal domain and a C-terminal repressor domain connected by a flexible linker [47]. The N domain is shared by all gasdermin family members and can bind with acidic lipids, including phosphatidylinositol phosphates (PIPs), phosphatidic acid (PA), phosphatidylserine (PS) and cardiolipin, which in turn form pores contained 16 symmetric protomers in plasma membrane [47, 48]. Intramolecular interaction between N-terminal and C-terminal fragments of gasdermins prevents activation of pore-forming activity of N-terminal domain and execution of pyroptosis, whereas proteolytic cleavage by inflammatory caspases, including caspase-1 and caspase-11, at the flexible linker between these two domains liberates the cytotoxic N-terminal domain to oligomerize in membrane and form large oligomeric pores where IL-1β and IL-18 are secreted [49, 50]. Structure-guided mutagenesis indicates that execution of pyroptosis is dependent on pore-forming activities of the gasdermin-N domain [47]. GSDMD is the first executor of pyroptosis to be discovered. GSDMD could be cleaved by caspase-1 and caspase-11 to trigger pyroptosis in macrophages [14, 21, 22]. GSDMD is the only caspase-1 substrate that induces pyroptosis. GSDMD-deficent cells resist to pyroptosis induced by activation of inflammasome. However, cells lacks of GSDMD are still susceptible to caspase-1-mediated cell death [51]. In the absence of GSDMD, activation of caspase-1 results apoptosis through activating caspase-3 and -7. During apoptosis, active caspase-3 and -7 inactivate GSDMD by cleaving GSDMD at Asp-87, thus blocking pyroptosis [52]. Murine GSDMD also could be cleaved at Asp-27 within an IPVD motif by caspase-7 [53]. Pore formation by GSDMD N-terminus is required for release of IL-1β and IL-18, but not essential for plasma membrane rupture during lytic cell death. It has been proposed that cells may repair the damage to cell membrane caused by the GSDMD-N, because of that formation of Gasdermin-N pore does not definitely leads to cell death [54]. For example, hyperactive macrophages release IL-1β through GSDMD pore, but keep alive [55]. Recently, a study suggests that formation of GSDMD pores is sufficient for inducing the maturation and release of IL-1α upon inflammasomes activation, which suggests that it may have an important role in settings without IL-1β [56].

In chemotherapy induced pyroptosis of epithelial cells, cleavage of GSDME at the linker is executed by active caspase-3 [16, 42]. TNF-α treatment and chemotherapy also induce pyroptosis in GSDME-expressing cancer cells via activation of caspase-3 [42]. Deletion of GSDME tends to disassemble into small apoptotic bodies upon activation of caspase-3. For example, lobaplatin treatment induces GSDME-mediated pyroptosis in colon cancer cells. However, knocking out GSDME switches cell death from pyroptosis to apoptosis, without affecting the cytotoxicity of lobaplatin on tumor growth and tumor formation of colon cancer cells [15]. Co-treatment of a PLK1 inhibitor BI2536 with cisplatin activates caspase-3/GSDME pathway and enhances the chemosensitivity of cisplatin in esophageal squamous cell carcinoma through induction of pyroptotic cell death [57]. Thus, gasdermin proteins, rather than caspases, act as the central molecule that switches apoptosis to pyroptosis upon death stimuli [16, 47]. In addition to caspase-3, killer cell or CAR T cell -derived GZMB also cleave GSDME at D270, unleashing its pore-forming activity to trigger caspase-independent pyroptosis in GSDME-positive cancer cells [44, 45]. Thus, gasdermin-mediated pyroptosis underlies the main killing mechanism of cytotoxic lymphocyte. Interestedly, GSDME-N not only forms pores in the plasma membrane, but also permeabilizes the mitochondrial membrane, leading to release of cytochrome c and augment caspase-3 activation and apoptosome. GSDME-deficient cells exhibit reduced cytochrome c release and caspase-3 activation upon intrinsic and extrinsic apoptotic stimuli. Like GSDME-N, GSDMD-N generated by inflammasome also permeabilizes the mitochondria [58]. Thus, cleavage of gasdermins links inflammasome activation to downstream activation of the apoptosome.

In addition to GSDMD and GSDME, N-terminal domain of GSDMA, GSDMA3, GSDMB, and GSDMC have all been proposed to form pores in membrane and execute pyroptosis [47]. GSDMB is specifically cleaved by lymphocyte-derived GZMA, unleashing its pore-forming activity and inducing pyroptosis in GSDMB-expressing cancer cells [43]. Caspase-8 activated by TNF-α cleaves GSDMC at it linker, liberating the GSDMC N-terminal domain to trigger pyroptosis in cancer cells [59].

The pore-forming activity of GSDME may be regulated by phosphorylation at a highly conserved Thr6 residue, because when Thr6 residue was replaced by glutamate (phosphomimetic), the pyroptotic activity of GSDME is significantly inhibited. Mechanistic study revealed that phosphorylation of Thr6 prevents GSDME-N dimerization/oligomerization in membranes but does not affect its membrane localization [58]. Similarly, phosphorylation of Thr8 of GSDMA which is equivalent to Thr6 in GSDME also causes complete abolishment of its pyrototic activity [58]. Recently, it has been demonstrated that palmitoylation on C-terminal of GSDME is required for pyroptosis induced by chemotherapy drugs. Palmitoylation of GSDME-C seems to dissociate the intramolecular interaction between N-terminal and C-terminal of GSDME protein, because 2-bromopalmitate treatment inhibits palmitoylation of GSDME-C and then promotes interaction between GSDME-C and GSDME-N [60].

### Pyroptosis exerts tumor suppressive function

Resistant to cell death is one of hallmarks of human cancers [1]. Apoptosis has been linked to tumor suppression and recognized as the major mechanism underlying anti-tumor therapeutic approaches, like radiation therapy (RT) and chemotherapy. As a non-lytic form of cell death, apoptosis is generally immunogenically silent. In contrast, lytic cell death, including necroptosis [61–64] and pyroptosis, is pro-inflammatory. Chronic inflammation is a well-known cancer-fueling process during cancer initiation and progression. It has been proposed that chronic inflammation increases the risk of cancer. Local inflammatory microenvironment is favorable tumor growth, angiogenesis, invasion, and metastasis [65]. Active necroptosis promotes intestinal inflammation in children with inflammatory bowel disease (IBD) and IBD mouse models [66], which is an inflammatory disease with enhanced risk for development of gastrointestinal malignancies. It has been shown that in vivo necroptosis is more efficient to induce antigen cross-priming [6]. However, it is still uncertain whether necroptosis promotes or restricts tumors initiation and progression [67]. Evidence showed that in vivo necrosome activation suppresses anti-tumor immunity through enhancing the infiltration of immune-suppressive myeloid cellular subsets, whereas deletion RIPK3 or inhibition RIPL1 in vivo enhances adaptive immunogenicity and prevents pancreatic oncogenesis in mice through repressing chemokine attractant CXCL1 and Mincle signaling [68], consistent with the pro-inflammatory properties of necroptosis and the cancer-promoting effects of inflammation. Inhibition of necroptosis by a specific chemical inhibitor Nec-1 also ameliorates inflammation and prevents colitis-associated tumorigenesis in a mouse model of inflammatory bowel disease [69]. In addition, necroptosis of endothelial cells promotes tumor cell extravasation and metastasis [70]. As a lytic, inflammatory type of cell death, pyroptosis leads to inflammation, which could increase the risk of cancer. However, it has been shown that Pycard^(−/−)^, Casp1^(−/−)^ mice and Nlrp3^(−/−)^ mice are prone to inflammation associated colon cancer [71, 72], suggesting that inflammasome activation or induction of pyroptosis restricts, rather than promotes, colon cancer development. A study indicates that genetic ablation of GSDMD mitigates the development of non-alcoholic steatohepatitis (NASH) [73]. NASH-related cirrhosis is associated with increased risk for liver cancer [74]. However, to date, there is no direct evidence suggests that deletion of would gasdermins family member affect spontaneous or induced cancer occurrence. Thus, the specific role of pyroptosis in tumorigenesis deserves further study. Studies suggested pyroptosis may function as a tumor suppression mechanism. Recent studies have proved that induction of pyroptosis in malignant cells provides an alternative approach to kill cancer cells. GSDME is silenced in most cancer cells [42]. Overexpression of wild type GSDME inhibits tumor growth in immunocompetent mice, whereas overexpression cancer associated GSDME mutants which lose the ability to execute pyroptosis fail to delay tumor growth [44]. Uncleavable GSDME (D270A) or pore-forming defective F2A nonfunctional mutants also fail to inhibit tumor growth, indicating tumor suppression by GSDME is dependent on its activity to execute pyroptosis [44]. It has been shown that mammalian STE20-like kinase 1 (MST1) is decreased in pancreatic ductal adenocarcinoma. Restored expression of MST1 in suppresses the proliferation, migration, invasion, and cell spheroid formation of pancreatic ductal adenocarcinoma cells through caspase-1–induced pyroptosis [75]. Thus, these studies suggest that pyroptosis represents a new way to eliminate cancer cells.

### Pyroptosis reprograms tumor microenvironments and evokes anti-tumor immunity

**Fig. 2:**
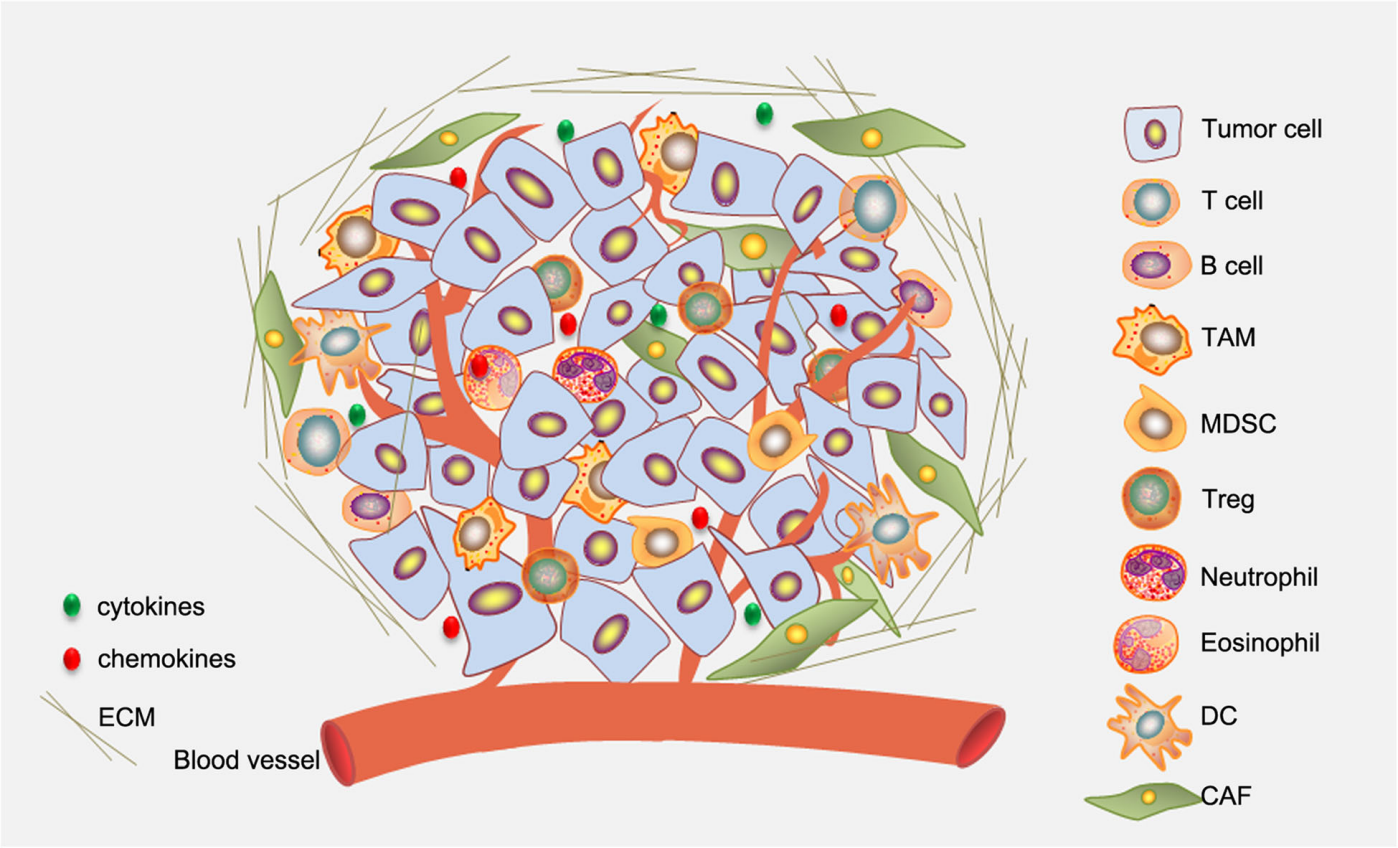
Immunosuppressive tumor microenvironments. Tumor microenvironments are composed of cellular components, extracellular matrix and interstitial fluid. Chemokines secreted from cancer cells recruit a variety of immune cells infiltrating into tumor. The interactions between cancer cells and the infiltrated immune cells determine the progression of cancer and therapeutic efficacy. Immune suppressive cells, including Treg, MDSC and M2 type TAM, limit function of the cytotoxic T cells and make tumor microenvironments immunosuppressive. Furthermore, tumor derived cytokines, like TGF-β, IL-6, etc., also suppress immune responses

Tumor microenvironments (TMEs) are composed of cellular components, extracellular matrix (ECM) and interstitial fluid. The cellular components of TME include tumor cells themselves, stromal cells (such as fibroblasts), endothelial cells of blood and lymphatic vessels, neuronal cells and infiltrating immune cells [76] (Fig. 2). The balance between pro-tumorigenic and anti-tumor factors in the microenvironment regulates tumor growth [77]. Cancer cells extensively communicate with stroma cells and convert them into allies from within, thus impacting diverse aspects of tumor biology [78, 79]. On the one hand, tumor cells recruit stromal cells, inflammatory or/and immune cells, etc. by actively secreting inflammatory factors, growth factors, extracellular matrix and its metabolites to stimulate angiogenesis and lymphogenesis. On the other hand, the stromal cells and inflammatory cells residing in TME have protection and supportive effects on the cancer cells. The interplay of tumor cells and non-tumor cells shapes the TME that allows tumor cells growth, invasion, and facilitates escaping the tumor immunosurveillance [78, 79]. Failure of tumor immunosurveillance causes the clinical appearance of cancer and cancer progression. Accumulating evidences indicate that TME plays a central role in the process of tumor occurrence, immune escape, progression and metastasis [80]. Immunotherapeutic approaches, including approved immune checkpoint inhibitors (ICIs) anti-CTLA-4, anti-PD-1/PD-L1 antibody and chimeric antigen receptor (CAR) T-cell therapy, have greatly improved clinical outcome in treating human cancer [81–83]. However, only a small portion of patients achieve durable benefit [84]. The efficacy of immune therapy relies on the pre-existing anti-tumor immunity, whereas is hampered by suppressive tumor immune microenvironment (TIME) that limits the ability of T cells to eradicate tumor cells [85–88]. Aberrant tumor vasculature generates a physical barrier for T cell trafficking. Otherwise, infiltration of immune suppressive cells, including regulatory T cell (Treg), myeloid derived suppressive cells (MDSC) and M2 type tumor associated macrophages (TAM), limits function of the cytotoxic T cells and makes solid tumor are refractory to immune-therapy [89–93] (Fig. 2). Furthermore, the TIME is characterized by hypoxia and low pH (*pH* < 4), which suppress the activity of cytotoxic T lymphocytes [94]. In addition, glucose-deprived whereas cholesterol enriched TIME further exacerbates T cell exhaustion [95–99]. Microenvironment normalization is prerequisite for initiating anti-tumor immunity [100]. In addition, vascular normalization not only promotes the delivery anti-tumor drugs, but also improves the immune cell infiltration [101]. The complexity of TIME has brought great obstacles to cancer treatment. Therefore, it is necessary to develop new strategies to ameliorate the suppressive TIME or normalize TIME [100, 102, 103].

**Fig. 3:**
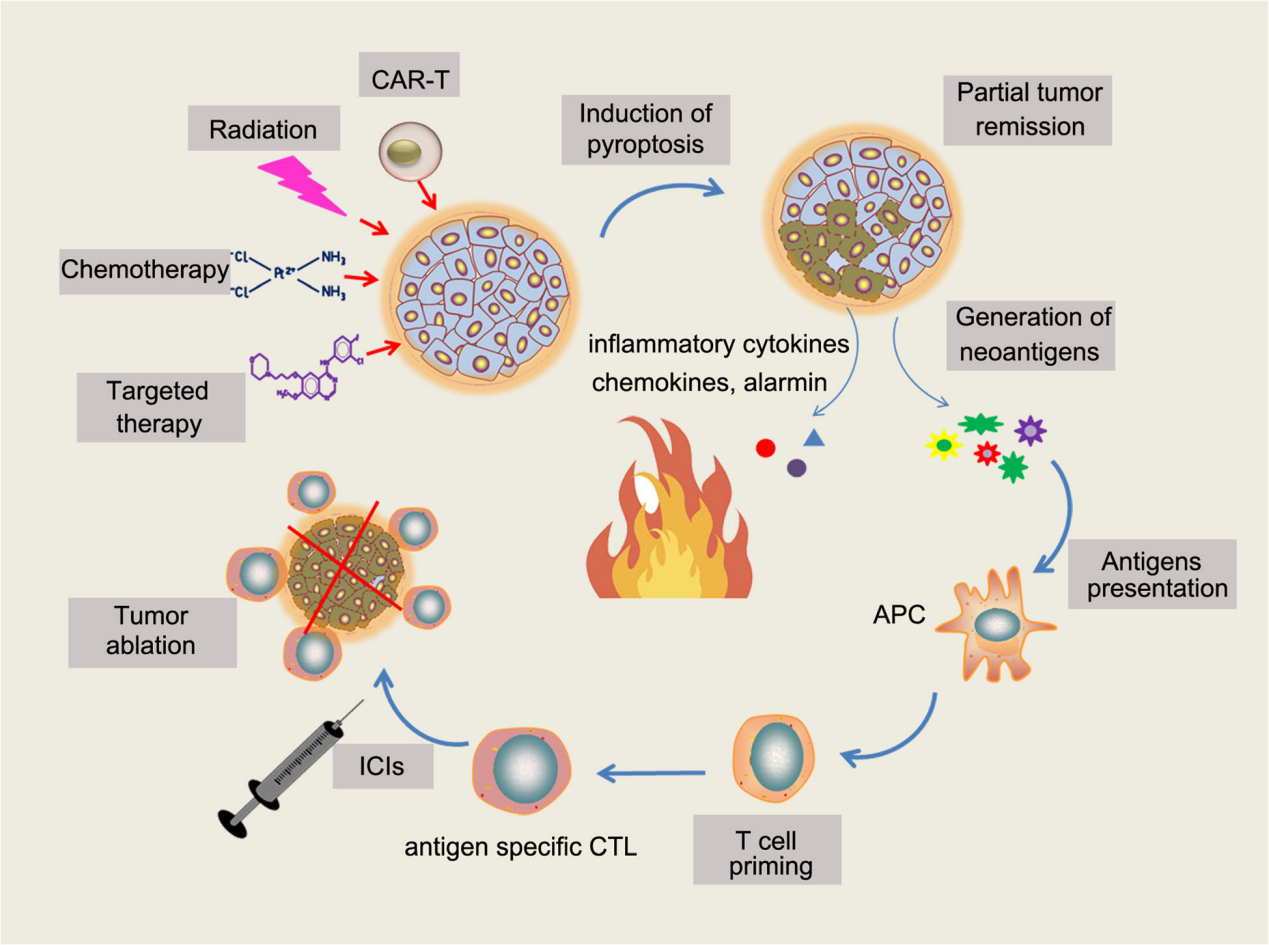
Induction of pyroptosis by therapeutic regimens evokes anti-tumor immune responses. Therapeutic modalities, including chemotherapy, targeted therapy, radiotherapy and CAR T cells, induce pyroptosis in cancer cells. Cancer cells undergoing pyroptotic cell death release pro-inflammatory factors (IL-1β, IL-18), alarmin (HMGB1, ATP, etc.), and causing intensive inflammation in the local environments. Pyroptosis in cancer generates abundant neoantigens, which are processed by antigen-presenting cells to promote the formation of antigen-specific cytotoxic T lymphocyte (CTL), thereby evoking anti-tumor immunity. Combination of pyroptosis-inducible therapeutic regimens with ICIs enhances anti-tumor immune responses and promotes tumor regression, achieving long-term control of cancer

Cell death plays a vital role in establishing adaptive immunity responding to microbial pathogen infection and transformed malignant cells [104]. Induction of immunogenic death of tumor cells is a reasonable strategy to establish a more immunologically active microenvironment, thus creating an opportunity to turn “cold” tumor to “hot” [105, 106] (Fig. 3). As a pro-inflammatory cell death form, induction of pyroptotic cell death of cancer cells provides an opportunity to overcome the immune desert phenotype of TME. It has been shown that tumors with high expression of wild type GSDME exhibit increased immune cell infiltration, including CD8^+^ T cells and natural killer (NK) cells, whereas GSDME-deficient tumors or tumors expressing loss-of-function GSDME mutants exhibit reduced immune cell infiltration [44]. Single-cell RNA sequencing revealed that pyroptosis-inducible therapy increases infiltration of CD4^+^, CD8^+^ T cells and natural killer cells, whereas reduces monocyte, neutrophil and myeloid-derived suppressor cell populations in experimental breast 4 T1 tumors. Furthermore, pyroptosis of 4 T1 tumor cells induces macrophage M1 polarization [107]. Importantly, tumor suppression effect of GSDME is abrogated in killer cytotoxic lymphocytes depleted mice or immune deficient mice, indicating tumor-suppressive function of GSDME requires pyroptosis-dependent activation of antitumor immunity [44]. In addition to directly eliminate tumor cells, induction of pyroptosis may overcome immunosuppression and reactivate a systemic anti-tumor immunity, which provides a great opportunity to achieve long-term control of cancer. As a major form of ICD, tumor cells undergoing pyroptosis generate large amounts of neoantigens that stimulate the systemic immune response to significantly hamper tumor progression [108] (Fig. 3). In addition, GSDME expression enhances anti-tumor adaptive immunity by promoting macrophage-mediated phagocytosis [44], which prevents immune evasion of tumor [109, 110].

### Therapeutic strategies to induce pyroptosis in cancer

Precise modulation of inflammasome activation and pyroptosis may provide great opportunity to improve the efficacy of immune therapy in the near future [111]. Recently, Wang et al. demonstrated that pyroptosis of less than 15% of tumor cells was sufficient to clear the entire 4 T1 mammary tumor graft. The degree of tumor regression is correlated with augmented anti-tumor immune responses and is absent in immune-deficient mice or upon T cell depletion [107], suggesting induction of pyroptosis is a powerful approach for cancer treatment.

### Induction of pyroptotic cell death by targeted therapy

It has been shown that inhibitors of the serine dipeptidases DPP8 and DPP9 (DPP8/9) activates the Nlrp1b inflammasome and induces pro-caspase-1 dependent pyroptosis in monocyte and macrophage [112, 113]. The effect of DPP8/9 inhibition to induce pyroptosis can be exploited to treat malignant cancer of myeloid origin. DPP8/9 inhibitors selectively induce pyroptotic cell death in human acute myeloid leukemia (AML) cells and inhibit human AML progression in mouse models, highlighting it’s a potential utility for AML treatment [114]. Specifically targeting KRAS, EGFR, or ALK mutants by small-molecule inhibitors elicit GSDME-mediated pyroptotic cell death in oncogenic mutations-driven lung cancer, pinpointing a previously unrecognized role of GSDME-dependent pyroptosis in molecular targeted therapy [115]. A latest study demonstrated that combinations of BRAF inhibitors and MEK inhibitors (BRAFi + MEKi) treatment, a FAD-approved approach for BRAF^V600E/K^-mutant melanoma, induce GSDME dependent pyroptosis and enhance cytotoxic T-cell infiltration. However, BRAFi + MEKi treatment loses the therapeutic effect against GSDME-deficient melanoma, indicating BRAFi + MEKi treatment kills melanoma cells mainly through induction of pyroptosis. More importantly, the efficacy of BRAFi + MEKi treatment on GSDME-expressing melanoma completely disappear in immune-deficient mice, suggesting that BRAFi + MEKi treatment eliminates melanoma cells through GSDEM-dependent anti-tumor immune responses [116].

### Induction of pyroptotic cell death by chemotherapy drugs

It has long been believed that apoptosis is the main form of chemotherapy-induced tumor cells death. However, recent progresses suggest pyroptotic cell death is a novel killing mechanism of conventional therapeutics including chemotherapy. For example, taxol treatment causes GSDMD-mediated pyroptosis in nasopharyngeal carcinoma, whereas suppression of pyroptosis has been proposed to be associated with taxol resistance in nasopharyngeal carcinoma [117]. Although cisplatin is widely believed to kill tumors by causing apoptosis, recent evidence suggests cisplatin induces lung cancer A549 cell through caspase-3/GSDME pathway. Silencing GSDME significantly attenuates the cytotoxicity of cisplatin against A549 cell [118]. Lobaplatin also induces GSDME-mediated pyroptosis by activating caspase-3 in nasopharyngeal carcinoma cells [119]. Combination of lose-dose cisplatin with PLK1 inhibitor BI2536 also induces GSDME-mediated pyroptosis in oesophageal squamous cell carcinoma cell lines [57]. Induction of sustainable anti-tumor immunity may improve the efficacy of cancer chemotherapy. Induction of GSDME-mediated pyroptosis evokes anti-tumor immunity, which enhances the ability of cisplatin to regress non-small cell lung cancer [120]. Our group found that the natural product triptolide exerts tumor suppression activity through inducing GSDME-mediated pyroptosis in head and neck cancer cells (unpublished data), highlighting its potential to serve as an adjuvant approach for cancer immune therapy. Mechanistically, displacement of hexokinase-II from mitochondria facilitates the release of cytochrome c and activation of caspase-3, which acts as up-stream events of GSDME-mediated pyroptosis upon triptolide treatment (unpublished data).

DNMTs mediated DNA hypermethylation of promoters represses transcription of gasdermins [121]. Zhao et al. developed a tumor-homing biomimetic nanoparticle (BNP) loaded with indocyanine green (ICG) and Decitabine (DCT), which can be photo-activated to induce pyroptotic death of cancer cells. DCT activates GSDME expression through reducing DNA methylation. Upon low-dose photo-activation, ICG in BNP causes extracellular calcium influx, leading to activation of caspase-3 and cleavage of GSDME. Thus, ICG and DCT in BNP synergistically promote cancer cell pyroptosis. Importantly, photo-activated pyroptosis induced by BNP not only directly eliminates primary tumor, but also enhances systemic antitumor immunity to suppress distant metastatic tumors. Thus, pyroptosis-inducible BNP is a novel approach to ameliorate immunosuppressive TIME and enhance the adaptive immunity in treating solid tumor [17]. Jin-Xuan Fan et al. developed chemotherapeutic nanocarriers combined decitabine with tumor-targeting nanoliposome loaded with cisplatin [122]. They demonstrated that administration of chemotherapeutic nanocarriers reactivates expression of GSDME and facilitates GSDME mediated pyroptotic cell death of tumor cells. This pyroptosis-based chemotherapy strategy enhances immunological effects of chemotherapy, reducing tumor growth, metastasis, and recurrence [122]. Thus, epigenetics-based tumor cell pyroptosis induced by chemotherapeutic nanocarriers provides an opportunity to enhance sensitivity to pyroptosis in cancers.

### Induction of pyroptotic cell death by radiation therapy and other physics therapy

Radiation therapy may release tumor antigens and may be an endogenous tumor vaccination event to create a proimmunogenic milieu stimulating local and systemic host cancer-specific immune responses [123]. It has been shown that radiotherapy elicits tumor-specific immune responses through promoting tumor infiltration of CD8^+^ T cells [124]. Local radiotherapy triggers ICD in cancer cells in a dose-dependent manner. Furthermore, radiation enhances chemotherapy-induced ICD in cancer cells [125]. Although there is limited literature describing pyroptosis of cancer cells directly induced by radiotherapy, it has been shown that radiation induces pyroptosis in bone marrow derived macrophages (BMDMs) [126]. Combinations of radiation regimens with immunotherapy are rational approaches to enhance anti-tumour immune responses and are actually used in clinical trials of a variety of human cancers [127–129]. Thus, it is needed to study whether pyroptosis is the major form of ICD caused by radiotherapy in vivo.

It has been shown that local treatment with high-frequency irreversible electroporation (H-FIRE) results in necrosis and pyroptosis in the mouse 4 T1 mammary tumor model, inducing a pro-inflammatory shift in the TME and enhancing cellular immunity. Local treatment with H-FIRE not only ablates the primary tumor, but also reduces metastatic lesions, which is dependent on the adaptive immune system [130]. Recently, Xiaorui et al. reported that cold atmospheric plasma, a novel promising anti-cancer treatment, induces GSDME-dependent pyroptotic cell death in GSDME-expressing tumor cells [131].

### Induction of pyroptotic cell death by immune therapy

Gasdermins-dependent pyroptosis elicited by granzymes underlies cytotoxic lymphocyte-killing mechanism [43, 44]. In addition to caspase-3, GzmB also cleaves GSDME protein at D270 to initiate pyroptosis in GSDME-expressing tumor cells [44]. Granzyme A (GZMA) from NK cells and cytotoxic T lymphocytes (CTLs) activates gasdermin B (GSDMB) in target cells. Introducing cleavable-GSDMB to mouse tumor cells improves tumor control by immune checkpoint therapy [43]. CAR T cells also induces target cell pyroptosis through release of granzyme B, activating caspase 3 and then resulting in cleavage of GSDME in B leukemic and other target cells [45]. Notably, the quantity of perforin/granzyme B in CAR T cells, rather than in existing CD8^+^ T cells, determines the activity of CAR T cells to induce target cell pyroptosis [45]. GSDMB expression is induced by interferon-γ (IFN-γ), thus it is reasonable to combined therapeutics of IFN-γ and immune checkpoint blockade to activate robust antitumour immunity [43]. Recently, Chengui et al. developed a tailored chimeric costimulatory converting receptor (CCCR) that comprised of the extracellular domain of PD1, transmembrane and cytoplasmic domains of NKG2D, and the cytoplasmic domain of 41BB. The CCCR-modified NK92 cells exhibit augmented activity against human lung cancer H1299 cells in vitro through induction of extensive pyroptosis [132]. However, another study argued that tumor suppression by antigen-specific primed cytotoxic T cells is independent of necroptosis or pyroptosis [133]. Thus, pyroptosis is an immune-stimulatory form of cell death and can synergize with immune checkpoint agents to improve the efficacy of immune therapy. Recently, metformin has been reported to induce pyroptosis in cancer cells [134, 135]. Given that metformin promotes antitumor immunity and improves efficacy of ICIs in malignant cancers [136–139], it is possibly that induction of pyroptosis of cancer cells by metformin may reprogram TIME toward “infiltrated-inflamed”.

### Inflammasome activation mediates therapy induced tissue damage

Conventional cancer treatments such as chemotherapy and radiotherapy tend to kill cells that are in a rapidly proliferating status, including rapidly growing cancer cells and normal cells (e.g., hematopoietic cells). Therefore, these conventional therapies often cause adverse side effects, including myelosuppression and reduced immunity, which may reduce the quality of life for patients and may potentially lead to treatment failure. For example, damage of hematopoietic stem and progenitor cells induced by chemotherapy results in multi-lineage myelosuppression [140]. Understanding the mechanisms underlying normal tissue injury caused by chemotherapy and radiotherapy is the basis for preventing these unwanted side effects. Recent evidence suggests that pyroptotic cell death of normal cells induced by therapeutic approaches plays an essential role in therapy-induced tissue damage and inflammation. During the past decades, apoptosis was considered to be the primary death form triggered by chemotherapy drugs. However, GSDME expression allows occurrence of pyroptosis upon death stimuli which originally induce apoptosis [47]. It has been shown that GSDME is widely expressed in normal tissues. Chemotherapy drugs activate pyroptotic cell death in GSDME-expressing cells through caspase-3-mediated cleavage of GSDME. GSDME-dependent pyroptosis largely contributes to chemotherapy drugs-induced tissue damage, because loss of GSDME ameliorate/mitigate chemotherapy related toxicity in mice [42]. A latest study suggested that suppression of inflammasome assembly prevents pyroptotic cell death of conventional dendritic cells (cDCs), thus making cDCs retain the ability to prime both CD4^+^ and CD8^+^ T cells [141]. Cisplatin is one of the most broadly used chemotherapy drugs. Nephrotoxicity is one of severe side effects caused by cisplatin. A latest study revealed that GSDMD-mediated pyroptosis in mouse kidney tissues and renal tubular epithelial cells may contribute to cisplatin-induced acute kidney injury. Deletion of GSDMD significantly ameliorate cisplatin-induced acute kidney injury in mice, whereas mice with GSDMD-N fragment overexpression in the kidney are more vulnerable to acute kidney injury caused by cisplatin [142]. Pyroptosis of cardiomyocytes is a plausible mechanism for severe cardiotoxicity caused by anti-tumor drugs [143]. Recently, Zheng et al. demonstrated that activation of Bnip3-caspase-3-GSDME pathway upon doxorubicin (Dox) treatment triggers GSDME-mediated pyroptosis, which is responsible for DOX-induced cardiotoxicity in vivo [144]. Dox treatment leads to hyper activation of NLRP3 inflammasome and pyroptotic cell death of cardiomyocytes, which underlies mechanism for dilated cardiomyopathy (DCM) occurred in Dox-treated heart tissues. Loss of either NLRP3 or caspase-1 protects mice from Dox-induced DCM [145]. These results suggest that targeting the inflammasome may help to control the adverse side effects induced by chemotherapy drugs.

It has been demonstrated that radiation could activate inflammasome in various immune cells, including macrophages, dendritic cells, NK cells, T cells, and B cells, in a dose-dependent manner. Knocking out caspase-1 significantly alleviate hematopoietic cell lose induced by radiation [146]. Radiation induced caspase-1 activation in immune cells is NLRP3-independent, but could be prevented by allopurinol treatment [146]. It has been shown that radiation induces pyroptotic cell death of BMDMs in vitro and in vivo through activating NLRP3 inflammasome [126]. Deletion of NLRP3 remarkably suppresses pyroptosis of BMDMs as well as IL-1β level. Additionally, knocking out NLRP3 protects mice from radiation induced death. Thus, inhibition of NLRP3 inflammasome mediated pyroptosis may provide an effective strategy to diminish radiation caused tissue injury. It has been shown that 5-androstenediol treatment significantly suppressed the radiation-induced activation of inflammasome-mediated pyroptosis by disrupting the interaction between AIM2 and ASC, leading to amelioration of myeloid suppression and radiation injury in mice [147]. Another study also revealed that radiation activates AIM2 inflammasome and pyroptosis in BMDMs, attributing to radiation-induced lung inflammation and fibrosis [148]. Clinically relevant high dose of radiation activates NLRP3 and AIM2 inflammasomes but not the NLRC4 inflammasome, causing GSDMD-dependent pyroptosis in bones and the spleen [149]. These studies suggest that inhibiting inflammasome-pyroptosis signaling has the potential to prevent the tissue damage by intense radiation regimens. In a latest study, Jun et al. reported that disulfiram, a drug for treating alcohol addiction, selectively inhibits GSDMD-dependent pyroptosis and IL-1β release upon LPS stimuli and protects mice from LPS-induced septic death. Mechanistically, disulfiram at nanomolar concentration allows IL-1β and GSDMD processing but abrogates pore formation of GSDMD-N through covalently modifying human/mouse Cys191/Cys192 in GSDMD, making it an attractive new therapeutic indication for repositioning this safe drug to counteract inflammation [150].

## Conclusion and future perspectives

Anti-tumor immunity not only prevents tumorigenesis, but also is prerequisite for the success of cancer immunotherapy. Growing evidences consistently suggest that pyroptosis-based therapeutic strategies could be combined with immunotherapy to improve the systemic control of cancer. Established tumors are typified with immunosuppressive TME, which reduces infiltration of T cells and restricts the anti-tumor activity of cytotoxic T cells. Philosophy of cancer biology has now been shifting from the tumor-cell-centric view to a microenvironment-centric paradigm. In order to achieve long-term success of cancer control, it is undoubtedly necessary to establish anti-tumor immunity by therapeutic approaches. Induction of pyroptosis of tumor cells has been proved to enhance immunogenicity of tumor and turn “cold” tumor to “hot” by attracting more anti-tumor lymphocytes. Recent studies have shown that conventional treatments, including chemotherapy and targeted therapy, reverse immunosuppressive microenvironments surrounding tumor cells and re-establish local or systemic anti-tumor immunity by inducing pyroptotic cell death of tumor cells [116]. In addition to directly kill tumor cells, chemotherapy and targeted therapy have the potential to induce local and systemic immune responses through inducing ICD. Induction of pyroptosis in tumor cells generates an antigen source for the restoration of antitumor immunity. It is deduced that the “restoration” of host antitumor immunity by chemotherapy, radiotherapy or targeted therapy is essential for complete tumor regression after administering treatment modalities [151–156]. On the other hand, both chemotherapy and radiotherapy may cause pyroptosis in immune cells or hematopoietic cells, resulting in impairment of anti-tumor immunity. In that case, it is vital to reduce side effects of conventional therapies by preventing pyroptotic cell death of immune cells induced by anti-cancer treatments. Intensive studies are needed to develop novel strategies that could specifically activate the pyroptosis in tumor cells but without damaging the immune system. To this end, specifically inducing pyroptosis in cancer cells by activating GSDME may provide a promising strategy to evoke anti-tumor immunity. Hypermethylation of promoter DNA silences the transcription of GSDME in human cancers. Future studies are needed to restore the expression of GSDME in tumor cells and develop specific GSDME agonists. Current clinical testing does not allow measurement of specific cell death types in vivo. Non-invasive molecular imaging methods that accurately determine forms of cell death should be developed.

## Data Availability

Not applicable.

## Abbreviations

RCD: Regulated cell death
PCD: Programmed cell death
ACD: Accidental cell death
ICD: Immunogenic cell death
IL-1β: Interleukin-1β
IL-18: Interleukin-18
DC: Dendritic cells
PAMPs: Pathogen-related molecular patterns
PRRs: Pattern-recognition receptors
LPS: Lipopolysaccharide
DAMPs: Damage-associated molecular patterns
NETs: Neutrophil extracellular traps
NINJ1: Ill-characterized nerve injury-induced protein 1
RT: Radiation therapy
IBD: Inflammatory bowel disease
MST1: Mammalian STE20-like kinase 1
TMEs: Tumor microenvironments
TIME: Tumor immune microenvironment
ECM: Extracellular matrix
Treg: Regulatory T cell
MDSC: Myeloid derived suppressive cells
TAM: Tumor associated macrophages
NK: Natural killer
BMDMs: Bone marrow derived macrophages
AML: Acute myeloid leukemia
H-FIRE: High-frequency irreversible electroporation
