# Correction to: Pyroptosis: a new paradigm of cell death for fighting against cancer

**DOI:** 10.1186/s13046-021-02020-7

**Published:** 2021-07-01

**Authors:** Yixin Tan, Quanzhu Chen, Xiaoling Li, Zhaoyang Zeng, Wei Xiong, Guiyuan Li, Xiayu Li, Jianbo Yang, Bo Xiang, Mei Yi

**Affiliations:** 1 NHC Key Laboratory of Carcinogenesis, Hunan Provincial Cancer Hospital and the Affiliated Cancer Hospital of Xiangya School of Medicine, Central South University, Tongzipo Road, Changsha, 410013 Hunan China; 2 The Key Laboratory of Carcinogenesis and Cancer Invasion of the Chinese Ministry of Education, Cancer Research Institute and School of Basic Medical Sciences, Central South University, Changsha, 410078 Hunan China; 3 Hunan Key Laboratory of Nonresolving Inflammation and Cancer, The Third Xiangya Hospital, Central South University, Changsha, 410013 Hunan China; 4 Department of Dermatology, The Second Xiangya Hospital, The Central South University, Changsha, 410011 Hunan China; 5 Department of Laboratory Medicine and Pathology, University of Minnesota, Minneapolis, MN 55455 USA; 6 Department of Dermatology, Xiangya Hospital, The Central South University, Changsha, 410008 Hunan China

**Correction to: J Exp Clin Cancer Res 40, 153 (2021)**

**https://doi.org/10.1186/s13046-021-01959-x**

Following publication of the original article [1], the authors identified minor errors in image-typesetting in Fig. 1; specifically the GzmA and GzmB labels had been transposed.

The corrected figure is given here. The correction does not have any effect on the results or conclusions of the paper. The original article has been corrected.

**Fig. 1:**
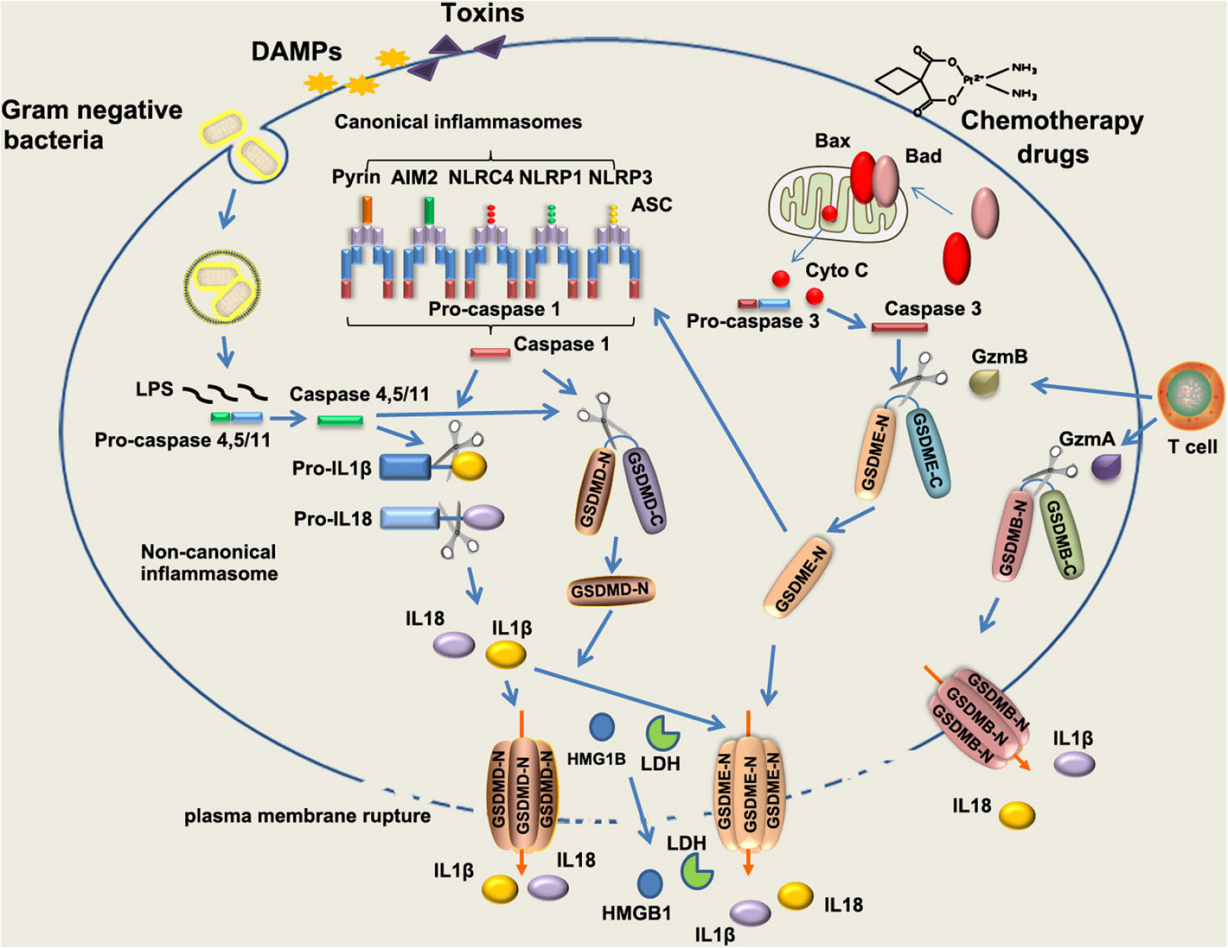
The canonical inflammasome and non-canonical inflammasome pathway in pyroptosis. The canonical inflammasome is assembled in response to exogenous pathogens and endogenous damage by intracellular sensor proteins, including NLRP1b, NLRC4, NLRP3, AIM2 and Pyrin. The canonical inflammasomes recruit pro-caspase 1 through inflammasome adaptor protein ASC, leading self-cleavage and activation of caspase 1. Active caspase 1 cleaves pro-inflammatory cytokines pro-IL-1β, pro-IL-18, leading to maturation of IL-1β, IL-18. Active caspase 1 cleaves GSDMD protein at the middle linker, liberating the cytotoxic N-terminus to form pore on plasma membrane, which allows the release of mature IL-1β, IL-18. In non-canonical pathway, LPS directly binds to murine pro-caspase 11 or its human homologs pro-caspase 4 and 5, leading activation of caspase 11/4/5. In non-canonical inflammasome pathway, cleavage of GSDMD is executed by active caspase 11 or caspase 4 and 5 upon direct binding of cytosolic LPS. Chemotherapy drugs could induce pyroptosis in epithelial cells through activating mitochondrial death machinery and caspase 3. In this case, GSDME is cleaved by active caspase 3. GSDME-N in turn activates NLRP3 inflammasome, leading to activation of caspase 1/GSDMD cascade, which promotes maturation of IL-1β, IL-18. Gasdermins could be cleaved by Lymphocyte-derived granzymes proteases, unleashing the pore-formation ability to trigger pyroptosis of cancer cells
