# Correction to: Pyroptosis: a new paradigm of cell death for fighting against cancer

**DOI:** 10.1186/s13046-021-02101-7

**Published:** 2021-09-22

**Authors:** Yixin Tan, Quanzhu Chen, Xiaoling Li, Zhaoyang Zeng, Wei Xiong, Guiyuan Li, Xiayu Li, Jianbo Yang, Bo Xiang, Mei Yi

**Affiliations:** 1 Hunan Key Laboratory of Cancer Metabolism, Hunan Cancer Hospital and the Affiliated Cancer Hospital of Xiangya School of Medicine, Central South University, Tongzipo Road, Changsha, 410013 Hunan China; 2 NHC Key Laboratory of Carcinogenesis, The Key Laboratory of Carcinogenesis and Cancer Invasion of the Chinese Ministry of Education, Cancer Research Institute and School of Basic Medical Sciences, Central South University, Changsha, 410078 Hunan China; 3 Hunan Key Laboratory of Nonresolving Inflammation and Cancer, The Third Xiangya Hospital, Central South University, Changsha, 410013 Hunan China; 4 Department of Dermatology, The Second Xiangya Hospital, The Central South University, Changsha, 410011 Hunan China; 5 Department of Laboratory Medicine and Pathology, University of Minnesota, Minneapolis, MN 55455 USA; 6 Department of Dermatology, Xiangya Hospital, The Central South University, Changsha, 410008 Hunan China

**Correction to: J Exp Clin Cancer Res 40, 153 (2021)**

**https://doi.org/10.1186/s13046-021-01959-x**

Following publication of the original article [1], the authors identified some minor errors in the Affiliation #1 and Affiliation #2 details. The correct details are as follows:

^1^ Hunan Key Laboratory of Cancer Metabolism, Hunan Cancer Hospital and the Affiliated Cancer Hospital of Xiangya School of Medicine, Central South University, Tongzipo Road, Changsha, 410013, Hunan, China.

^2^ NHC Key Laboratory of Carcinogenesis, The Key Laboratory of Carcinogenesis and Cancer Invasion of the Chinese Ministry of Education, Cancer Research Institute and School of Basic Medical Sciences, Central South University, Changsha 410078, Hunan, China.

The correction does not have any effect on the results or conclusions of the paper. The original article has been corrected.
